# Supplementary material for: Distinct Mechanistic Behaviour of Tomato CYP74C3 and Maize CYP74A19 Allene Oxide Synthases: Insights from Trapping Experiments and Allene Oxide Isolation
Source: Int J Mol Sci. 2023 Jan 23;24(3):2230. doi: 10.3390/ijms24032230 (PMC9916873; doi:10.3390/ijms24032230)
Supplement: Supplementary file 1 [file ijms-24-02230-s001.zip › ijms-2123487-supplementary.pdf]

Supplementary Results

Ethanol trapping experiments with 13-HPOD. Major products

Both LeAOS3 and ZmAOS preferentially utilized 9-HPOD. Brief incubations with 13-HPOD resulted in significantly lower product yields. Nevertheless, these trapping experiments were performed too. The results of 13-HPOD incubations with LeAOS3 and ZmAOS, followed by EtOH trapping, are presented in Supplementary Figures 1A and 1B, respectively. The presence of prominent peaks of 13-HOD (Me/TMS), 13-KOD (Me), and the epoxyalcohol, 11-hydroxy-12,13-epoxy-9-octadecenoic acid, indicated poor utilisation of the substrate by both enzymes. During the GC analyses, these compounds form a typical array of thermal isomerization of fatty acid hydroperoxides (Me/TMS). Nevertheless, the AOS conversion products were present, although less prominent than the peak of 13-HOD. First of all, this was the ethanolysis product **5** (Me), which was present in the GC-MS product profiles of both enzymes (Supplementary Figures 1A, B). The mass spectrum of product **5** (Me) and mass fragmentation scheme are presented in Supplementary Fig. 1C and its inset, respectively. It should be noted that the α−ketol (Me/TMS, the mass spectral data are not presented) was present in LeAOS3 trapping products (Supplementary Figure 1A) but not in ZmAOS products (Supplementary Figure 1B).

Supplementary Figures

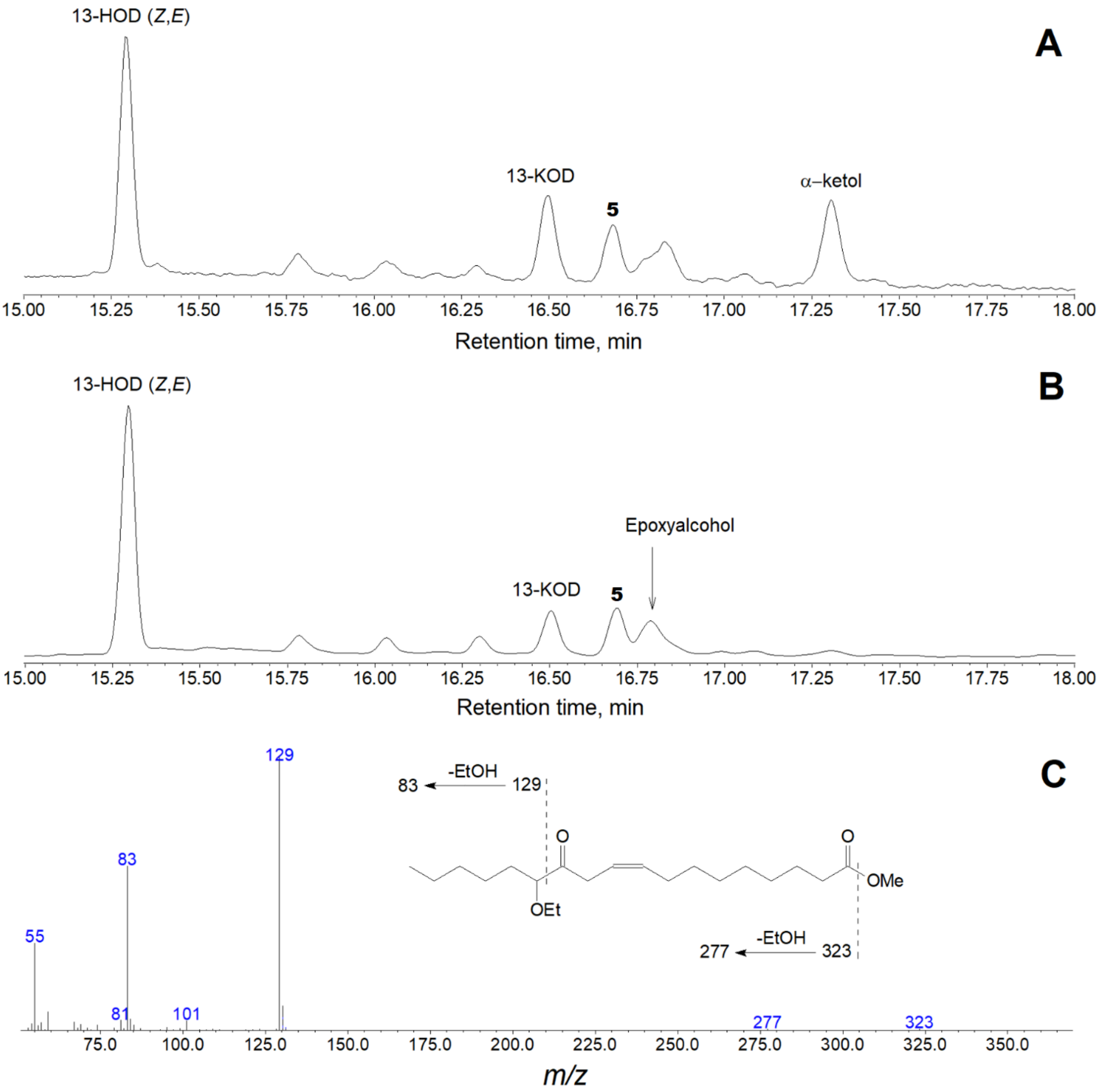

Supplementary Figure S1. Products of ethanol trapping experiments after the brief incubations of 13-HPOD with LeAOS3 or ZmAOS. The total ion current GC–MS chromatograms of products (Me/TMS) of biphasic (hexane/water 40:1 by volume, 60 s, 0 ºC) incubations of LeAOS3 (**A**) and ZmAOS (**B**) with 13-HPOD followed by (1) water freezing, (2) direct addition of ethanol excess to the hexane phase, (3) solvent evaporation, methylation and trimethylsilylation of products, and (4) GC-MS analyses of product derivatives. (**C**) The electron impact mass spectrum of the trapping product **5**. Inset, the mass fragmentation scheme. The detailed experimental conditions are described in the Materials and Methods.

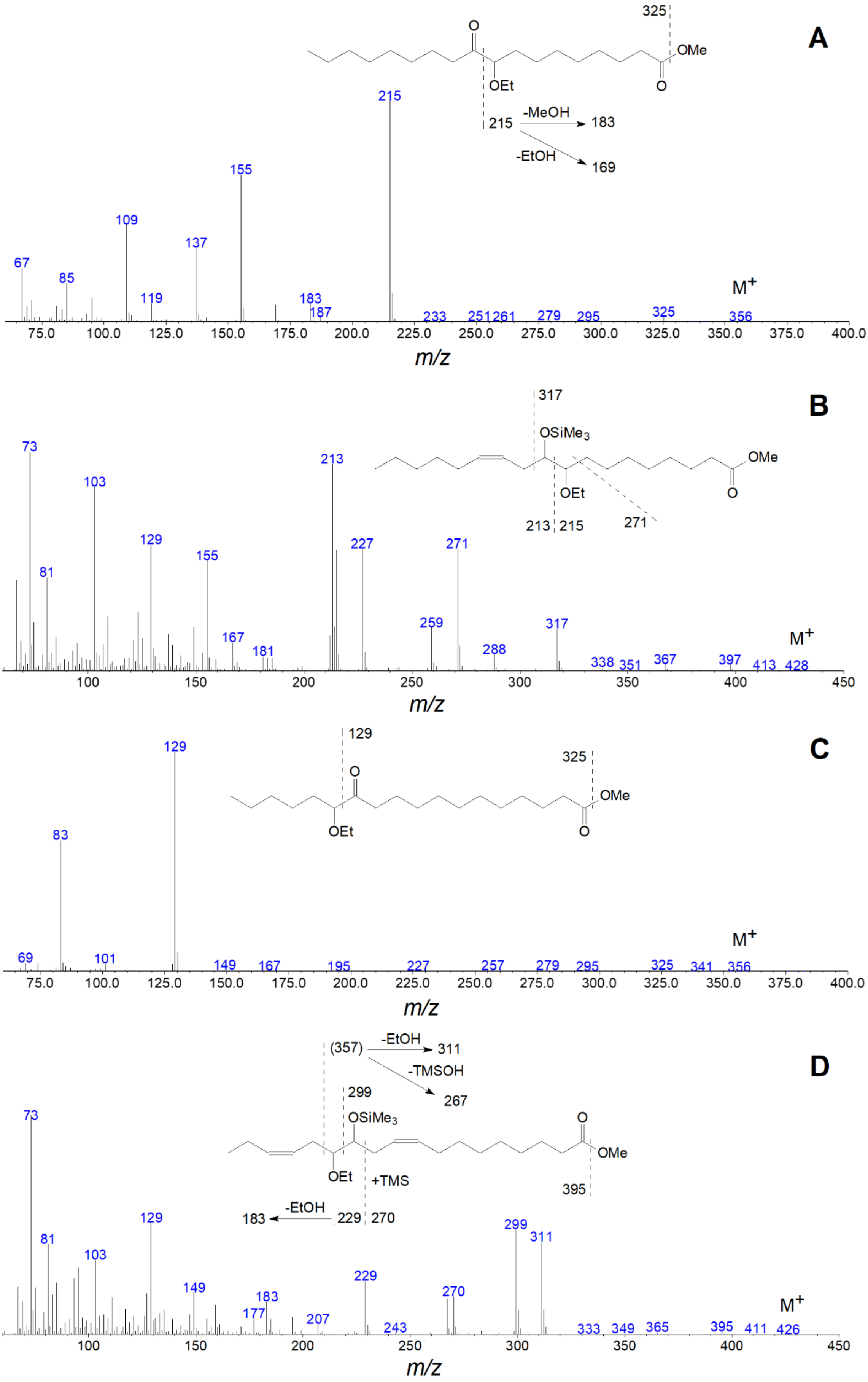

Supplementary Figure S2. The electron impact mass spectra of derivatives of trapping products **1** and **3** (**A**) hydrogenated product **1** (Me); (**B**) $NaBH_4$-reduced product **1** (Me/TMS); (**C**) hydrogenated product **3** (Me); (**D**) $NaBH_4$-reduced product **3** (Me/TMS).

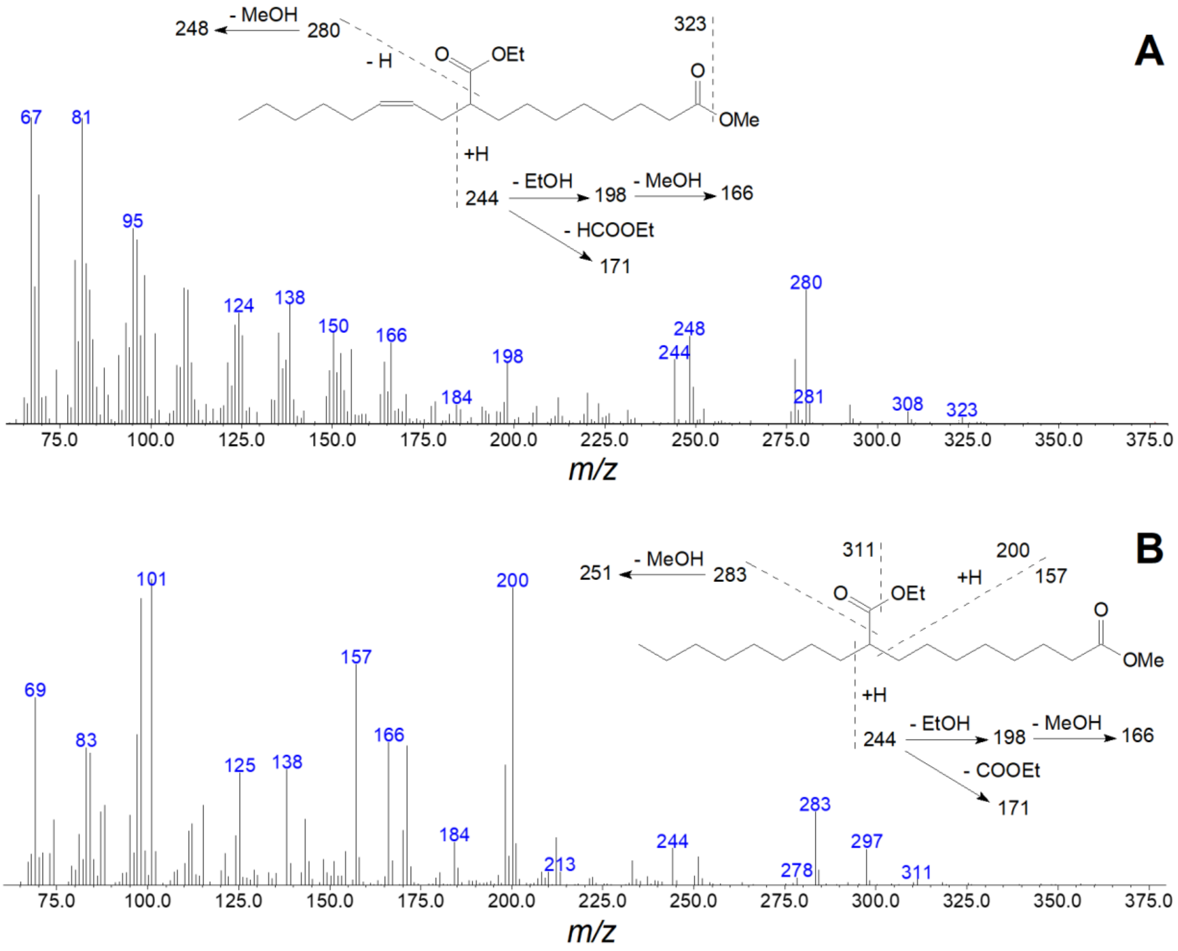

Supplementary Figure S3. The electron impact mass spectra of methyl esters of product **2** (**A**) and its hydrogenation product (**B**). Product **2**, the Favorskii-type rearrangement product formed via the cyclopropanone ethanolysis, was detected in addition to the allene oxide ethanolysis product **1** after the biphasic incubation of LeAOS3 with 9-HPOD followed by ethanol trapping. Insets, the mass fragmentation schemes.

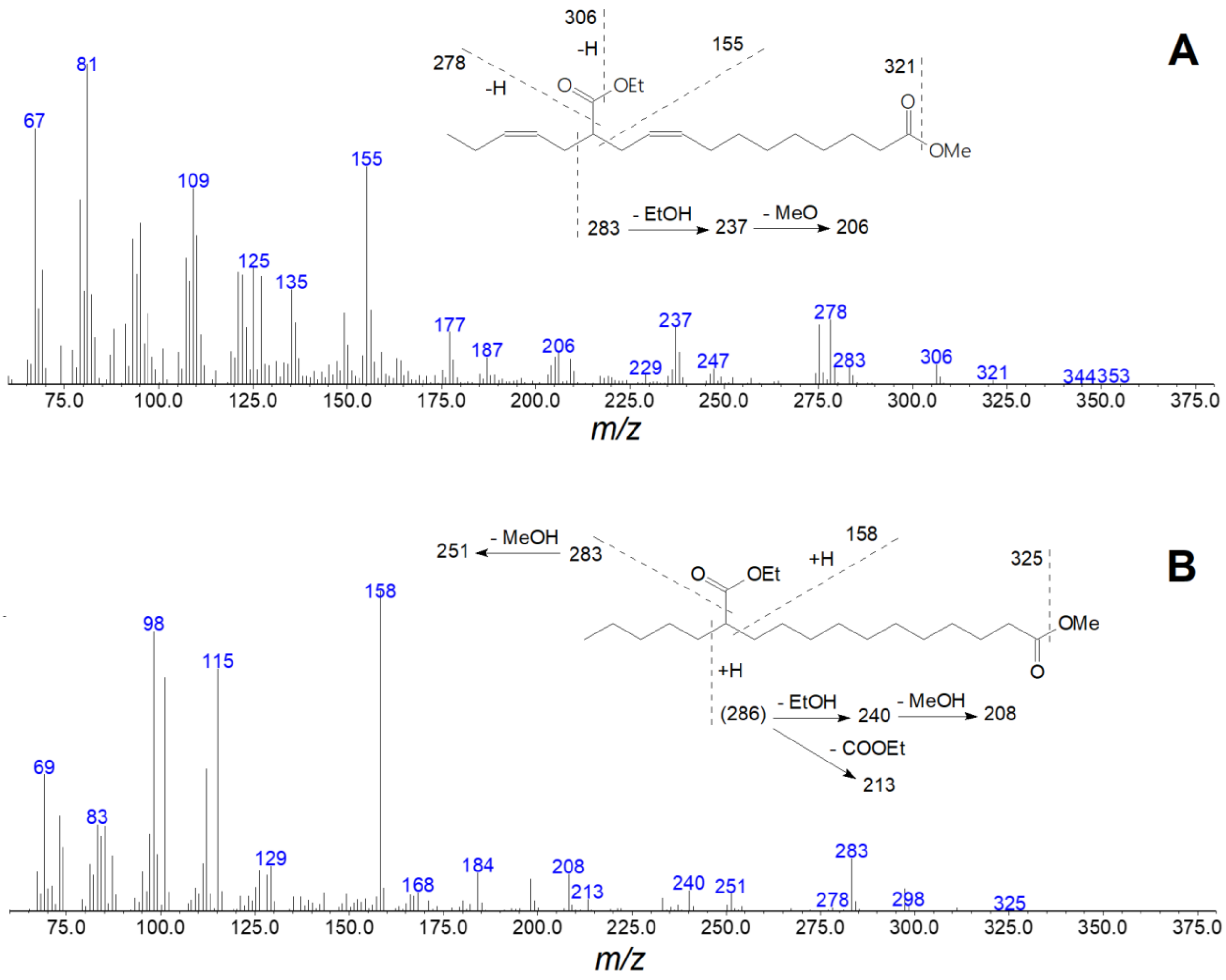

Supplementary Figure S4. The electron impact mass spectra of methyl esters of product **4** (A) and its hydrogenation product (B). Product **4**, a product of cyclopropanone ethanolysis, was detected in addition to the allene oxide ethanolysis product **3** after the biphasic incubation of LeOAS3 with 13-HPOT followed by ethanol trapping. Insets, the mass fragmentation schemes.

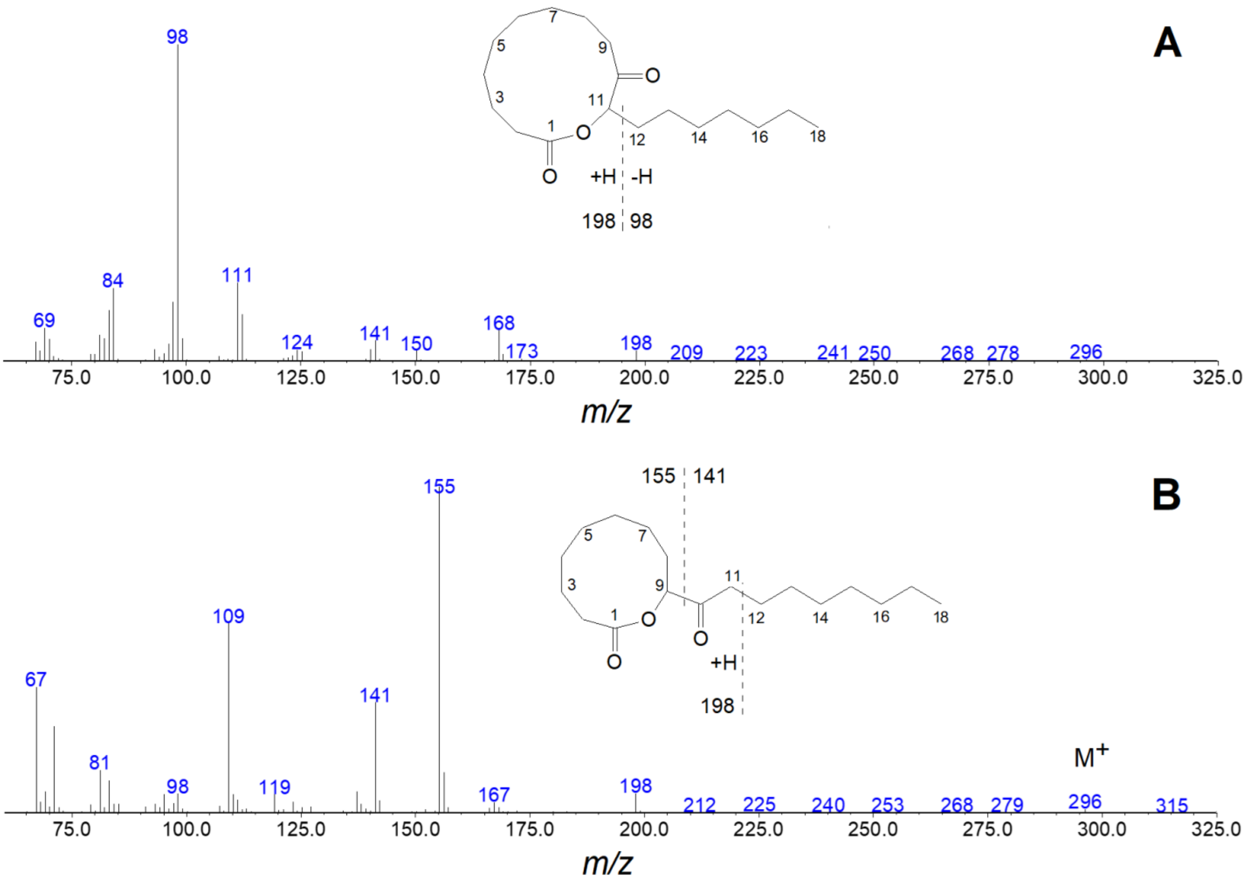

Supplementary Figure S5. The electron impact mass spectra of hydrogenated products **6** (**A**) and **7** (**B**) formed when 9,10-EOD was kept for 2 hours in hexane solution. Insets, the mass fragmentation schemes. The detailed experimental conditions are described in the Materials and Methods.

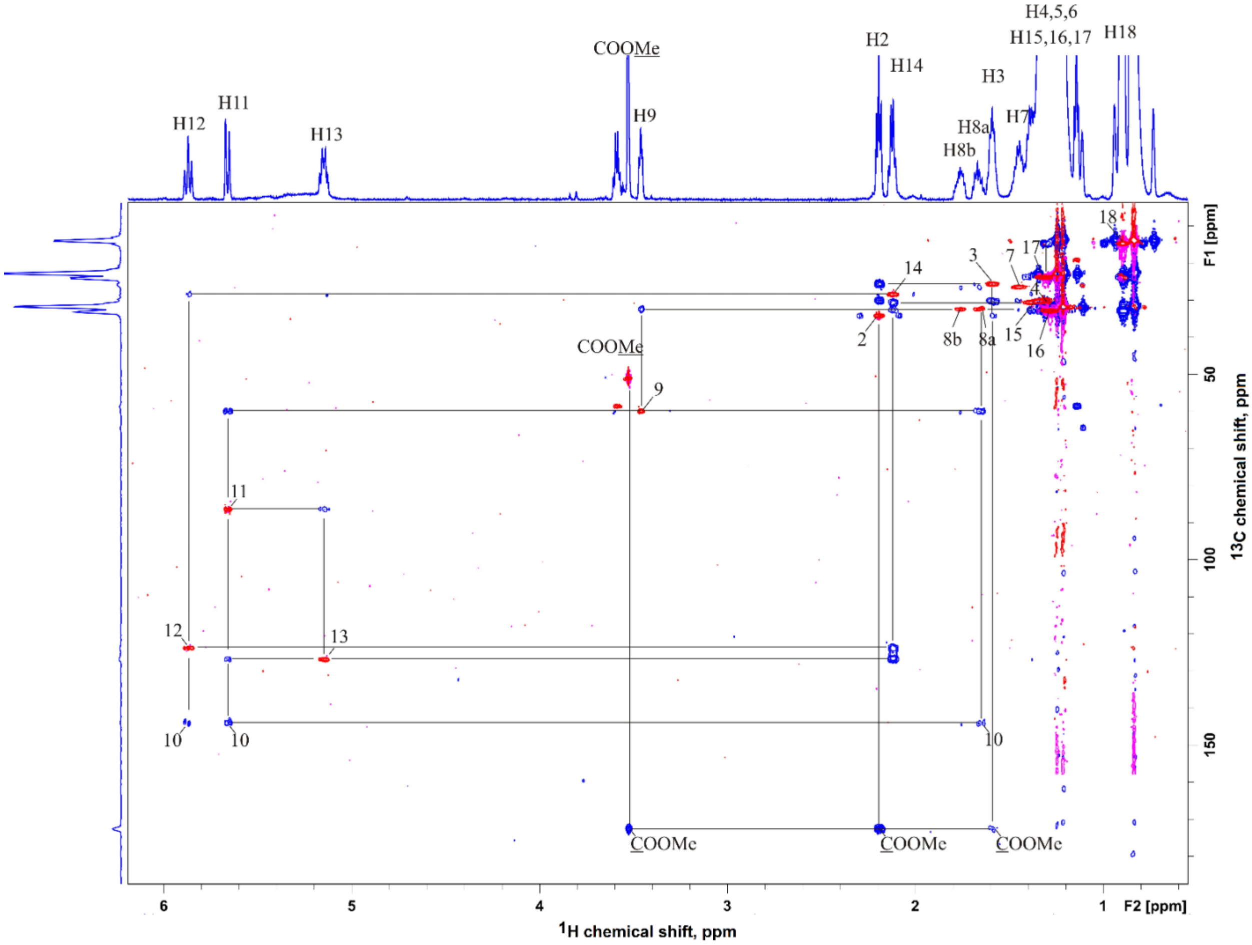

Supplementary Figure S6. The superimposed ^{1}H,^{13}C-HSQC (red) and ^{1}H,^{13}C-HMBC (blue) spectra ([$^{2}H_{14}$]n-hexane, 253 K) of allene oxide 9,10-EOD isolated and purified by NP-HPLC after the biphasic incubation of 9-HPOD with recombinant ZmAOS. Details of allene oxide preparation and spectral records are described in Materials and Methods. The spectral parameters are presented in Figure 7.

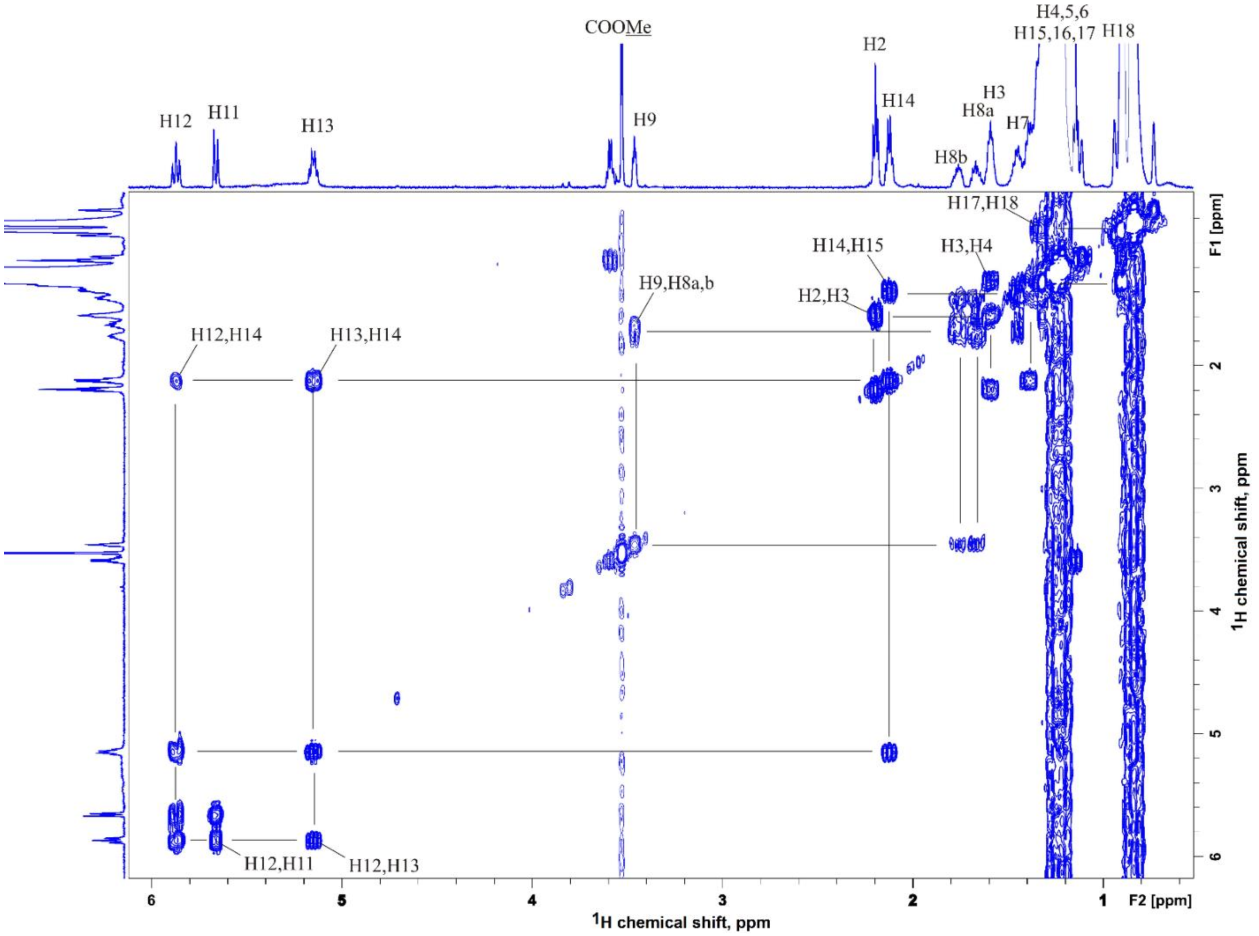

Supplementary Figure S7. The ^{1}H,^{1}H-COSY spectrum ([$^2H_{14}$]n-hexane, 253 K) for allene oxide 9,10-EOD purified by NP-HPLC after the biphasic incubation of 9-HPOD with recombinant ZmAOS. Details of allene oxide preparation and spectral records are described in Materials and Methods. The spectral parameters are presented in Figure 7.

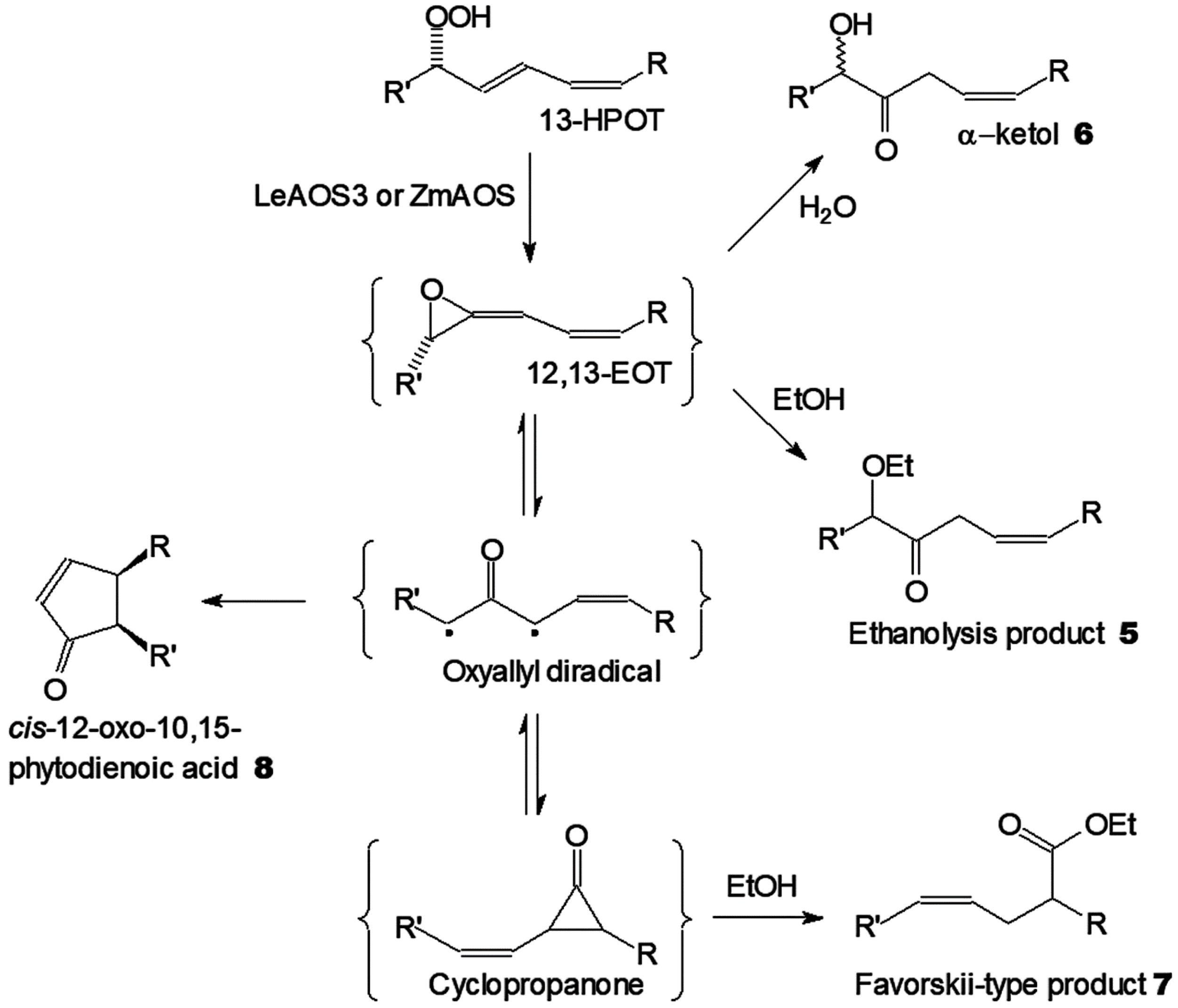

Supplementary Figure S8. Scheme of 13-HPOT conversions to various products in the presence of LeAOS3 or ZmAOS followed by ethanol trapping. R = -$(CH_2)_7COOH$; R′ = -$CH_2CH{=}CHCH_2Me$.

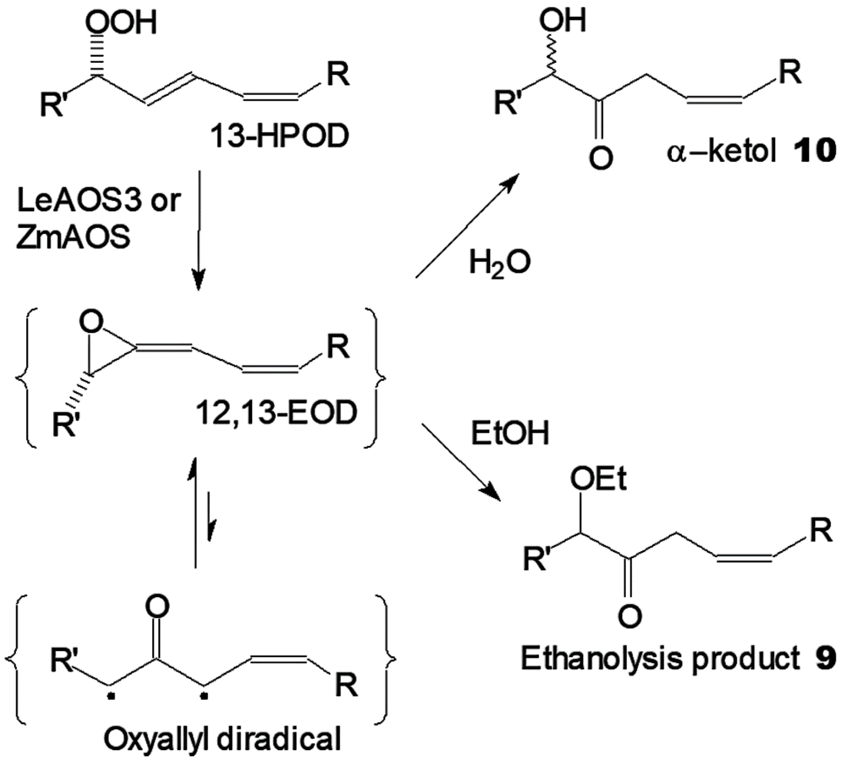

Supplementary Figure S9. Scheme of 13-HPOD conversions to various products in the presence of LeAOS3 or ZmAOS followed by ethanol trapping. R = -$(CH_2)_7COOH$; R′ = -$(CH_2)_4Me$.
